# Implementing school-based nutrition interventions in low- and middle-income countries: a scoping review of implementation strategies, frameworks, and outcomes

**DOI:** 10.1080/16549716.2026.2727312

**Published:** 2026-09-08

**Authors:** Oleksandr Kamyshnyi, Iryna Halabitska, Pavlo Petakh, Sanju Bhattarai, Abhijit Sen

**Affiliations:** a Department of Microbiology, Virology, and Immunology, I. Horbachevsky Ternopil National Medical University, Ternopil, Ukraine; b Department of Therapy and Family Medicine, I. Horbachevsky Ternopil National Medical University, Ternopil, Ukraine; c Department of Biochemistry and Pharmacology, Uzhhorod National University, Uzhhorod, Ukraine; d Institute for Implementation Science and Health, Kathmandu, Nepal; e Department of Public Health and Nursing, Faculty of Medicine and Health Sciences, Norwegian University of Science and Technology, Trondheim, Norway; f Center for Oral Health Services and Research (TkMidt), Trondheim, Norway

**Keywords:** School-based nutrition, implementation strategies, implementation outcomes, low- and middle-income countries, children and adolescents

## Abstract

School-based nutrition interventions are widely used to improve nutrition among children and adolescents in low- and middle-income countries. This scoping review examined implementation strategies, theories, models, frameworks, and implementation outcomes reported for school-based nutrition interventions in these settings. We included English-language primary studies conducted in school settings in low- and middle-income countries if they reported implementation strategies, implementation outcomes, theories, models or frameworks, barriers, facilitators, adaptation, sustainability, scale-up, or implementation processes. PubMed and Scopus were searched, and reference lists of included studies and relevant reviews were screened. Records were screened independently by two reviewers, and data were synthesized descriptively and thematically. The search identified 1,985 records, and eight studies met the inclusion criteria. The studies were conducted in Nepal, Ghana, Pakistan, Tanzania, and India. Implementation strategies included teacher-led delivery, training, stakeholder and community engagement, external partnership support, and integration into school systems. Reported barriers included limited funding, inadequate infrastructure, staff workload, weak coordination, limited monitoring, and dependence on external or parent contributions. Separately, implementation reporting was incomplete, particularly for reach, fidelity, and sustainability. Because the search was restricted to two databases and English-language publications, some relevant evidence may have been missed. Future studies should report implementation processes and outcomes more clearly to support adaptation, scale-up, and long-term delivery.

## Paper Context

- **Main findings:** Several included studies reported school-based nutrition interventions as feasible or acceptable, while also identifying barriers related to unstable funding, limited infrastructure, staff workload, poor coordination, limited monitoring, and dependence on external or parent contributions.
- **Added knowledge:** This review shows that implementation strategies, frameworks, reach, fidelity, and sustainability are not reported in enough detail in studies of school-based nutrition interventions in low- and middle-income countries.
- **Global health impact for policy and action:** The reviewed evidence identifies financing, infrastructure, staff training, coordination, monitoring, and community engagement as priorities for further implementation research and context-specific programme planning.

## Background

Malnutrition among children and adolescents in low- and middle-income countries (LMICs) remains a major challenge for health, educational attainment, and long-term human capital development [1]. Many LMICs are now experiencing a double burden of malnutrition, in which undernutrition coexists with overweight, obesity, and diet-related non-communicable diseases within the same populations, households, or communities [2]. Addressing this burden requires not only effective nutrition interventions but also evidence on how such interventions can be delivered, accepted, adapted, and maintained in routine school settings.

School-based nutrition interventions are increasingly recognized as a practical platform for responding to this double burden [3]. Schools provide regular access to large numbers of children and adolescents and create opportunities to combine food provision, micronutrient supplementation, nutrition education, and changes to the school food environment [4]. In this sense, school-based nutrition interventions may function as double-duty actions when they are designed or implemented in ways that can contribute simultaneously to the prevention of undernutrition, overweight, obesity, and diet-related disease.

School-based nutrition interventions include a broad variety of approaches, such as school meals and snacks, school feeding modifications, micronutrient supplementation or fortification delivered through schools, nutrition education, healthy canteen initiatives, and food environment or policy interventions. Research has shown that school-based food policies and programmes can address around 40% of the drivers of the double burden of malnutrition [5]. These interventions are relevant not only to health improvement but also to educational participation, social protection, and equity, particularly in resource-constrained settings where schools may be one of the few stable platforms for reaching children and adolescents at scale.

However, the impact of school-based nutrition interventions depends not only on whether they are effective in principle but also on how they are implemented in routine school settings. Implementation processes influence whether interventions are adopted by schools, implemented with adequate quality and reach, adapted to local needs, accepted by stakeholders, and sustained over time [6]. In LMICs, these processes are often influenced by limited funding, weak infrastructure, teacher workload, institutional capacity, policy environments, and coordination between education, health, agriculture, and social protection sectors [7].

Although the evidence base on school-based nutrition interventions has grown, implementation evidence remains fragmented [8]. Previous reviews have commonly examined nutritional, behavioural, educational, or obesity-related effects of school-based interventions [9,10], whereas implementation processes and outcomes have received comparatively less comprehensive synthesis [11,12]. Where implementation is addressed, studies often use different terminology, report different outcomes, or provide limited information on implementation strategies, theoretical frameworks, contextual determinants, and sustainability.

This represents an important gap for policymakers and programme implementers. Decisions about adaptation, scale-up, and long-term investment require more than evidence that an intervention can work under study conditions. They also require an understanding of what strategies support delivery, which contextual factors enable or constrain implementation, which frameworks guide implementation and evaluation, and which implementation outcomes are measured across studies.

Implementation science helps examine these issues by focusing on how interventions are adopted, carried out, adapted, and sustained in real-world settings. Concepts such as reach, acceptability, feasibility, adoption, fidelity, and sustainability help clarify whether interventions are being implemented as intended and whether they are likely to be maintained or scaled [7]. Implementation frameworks can also help organize evidence on barriers, facilitators, strategies, and mechanisms, making findings more useful for programme design and policy decision-making [13]. Unlike prior effectiveness reviews [14], the present review aims to map a broader set of implementation constructs, including strategies, outcomes, frameworks, adaptation, sustainability, and scale-up across diverse study designs.

This scoping review summarizes evidence on the implementation of school-based nutrition interventions designed to improve the nutritional status of children and adolescents in LMICs. Specifically, it addresses three questions: what implementation strategies are used to support the adoption, delivery, and sustainability of such interventions; which theories, models, methods, or frameworks are employed to guide implementation and evaluation; and which implementation outcomes are assessed and reported.

## Methods

This scoping review followed the Preferred Reporting Items for Systematic Reviews and Meta-Analyses extension for Scoping Reviews (PRISMA-ScR). Prior to commencing the review, we registered the review protocol on the Open Science Framework (OSF) (http://doi.org/10.17605/OSF.IO/QCXFZ).

### Study selection and eligibility criteria

We included English-language primary studies conducted in primary or secondary school settings in countries classified as low- or middle-income according to the World Bank classification used at the time of screening. Eligible studies examined a school-based nutrition intervention or programme and reported at least one implementation-related component, including implementation strategies, implementation outcomes, theories, models or frameworks, barriers and facilitators, adaptation, sustainability, scale-up, or intervention delivery processes. Anthropometric, dietary, nutrition knowledge, or behavioural outcomes were not required for inclusion unless they were reported together with eligible implementation-related evidence.

The school-based nutrition interventions included school meal, snack, or feeding programmes; school nutrition education, school food environment enhancement interventions including healthy canteen or food policy initiatives; and multicomponent programmes with nutrition components.

Eligible study designs included qualitative, quantitative, and mixed-methods studies, as well as cross-sectional studies, randomized controlled trials, quasi-experimental studies, and uncontrolled pre–post studies. Studies were eligible regardless of publication year if they reported implementation strategies, implementation outcomes, barriers and facilitators, or implementation-related findings for school-based nutrition interventions. Reviews, including systematic reviews, narrative reviews, scoping reviews, and other evidence syntheses, were excluded as units of analysis. However, their reference lists were screened to identify additional eligible primary studies.

Studies reporting the use of implementation strategies to support the adoption, implementation, sustainability, or scale-up of school-based nutrition interventions were included. Implementation strategies were defined as planned actions or methods used to enhance the uptake, delivery, and maintenance of an intervention, following the Expert Recommendations for Implementing Change (ERIC) taxonomy [15]. In addition, studies assessing implementation outcomes and barriers and facilitators to implementation were included. Implementation outcomes were defined as indicators of how well an intervention was implemented, rather than indicators of intervention effectiveness, following Proctor et al. [13]. These outcomes included acceptability, adoption, feasibility, fidelity, reach, and sustainability. Acceptability referred to the extent to which the intervention was considered suitable or satisfactory by students, teachers, parents, school staff, or programme implementers. Adoption referred to whether schools, teachers, or other implementers decided to use or deliver the intervention. Feasibility referred to whether the intervention could be delivered in the school setting with available resources, time, infrastructure, and personnel. Fidelity referred to whether the intervention was delivered as planned. Reach referred to the extent to which the intervention reached the intended students, schools, or stakeholder groups. Sustainability referred to whether the intervention could be maintained over time within routine school or programme systems. Reports that focused primarily on intervention effectiveness or nutritional outcomes were excluded if they did not provide sufficient standalone information on implementation strategies, implementation outcomes, barriers, facilitators, adaptation, sustainability, scale-up, or intervention delivery processes relevant to the review questions. When several publications described the same intervention, each report was assessed separately, and only reports containing eligible implementation-related evidence were included in the synthesis.

Studies conducted outside primary or secondary school settings and studies conducted in high-income countries were excluded. Interventions focused on clinical nutrition therapy or sports nutrition were also excluded. In addition, protocols, editorials, commentaries, opinion papers, conference abstracts with inadequate data reporting, and grey literature were excluded.

### Data sources and search strategy

A structured electronic search was conducted in PubMed and Scopus to identify relevant studies. The search strategy was developed by the review team using key concepts derived from the inclusion criteria. Search terms within each concept were combined using the Boolean operator ‘OR’, while concepts were combined using ‘AND’. The full search strategy is provided in Supplementary File 1. Searches in PubMed and Scopus were conducted between 1 March and 11 March 2026. The English-language restriction was applied during title and abstract screening and confirmed during full-text assessment. Additionally, the reference lists of studies included in the final review, as well as the relevant systematic reviews, were manually screened to identify additional eligible primary studies. PubMed and Scopus were selected because they provide broad coverage of biomedical, public health, nutrition, and multidisciplinary research. Grey literature was not included because the review was restricted to peer-reviewed primary studies with sufficient methodological and implementation reporting. For reports that could not initially be retrieved, we searched institutional library holdings, journal websites, PubMed, Google Scholar, and available open-access repositories. Reports that remained unavailable after these attempts were classified as not retrieved.

**Figure 1.**
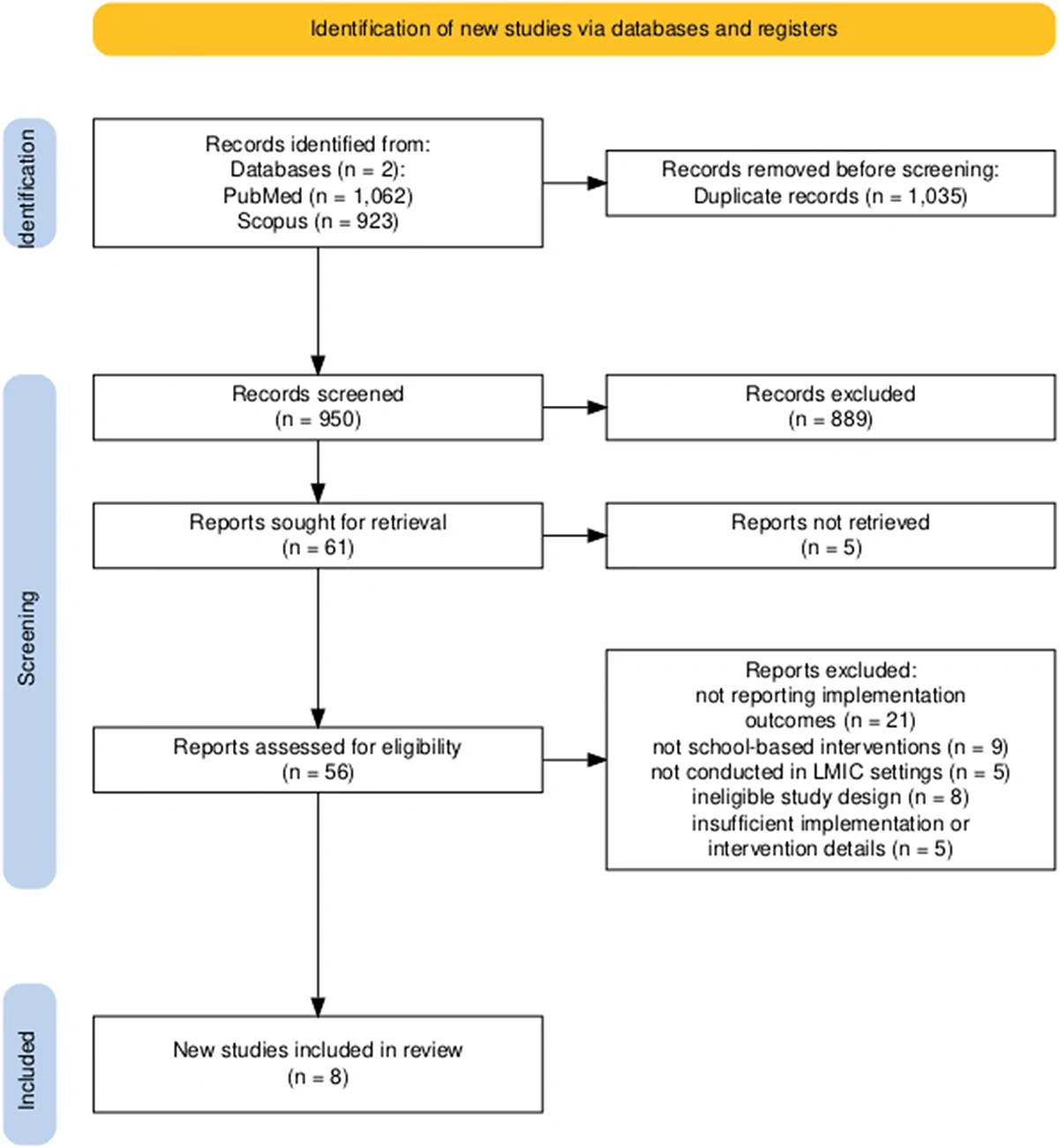
PRISMA flow diagram of study selection process.

All records identified through the database searches were imported into EndNote, and duplicates were removed before screening. Screening decisions were recorded in a standardized Excel-based screening sheet. Study selection was conducted in two stages. First, titles and abstracts were screened independently by two reviewers (P.P. and I.H.) against the predefined eligibility criteria. Full-text articles identified as potentially eligible were then assessed independently by the same two reviewers (P.P. and I.H.) to determine final inclusion. Disagreements at either stage were resolved through discussion and, when necessary, consultation with a third reviewer (O.K.). The study selection process, including reasons for exclusion at the full-text stage, was documented and presented using a PRISMA flow diagram (Figure 1).

### Data extraction

Data were extracted independently by two reviewers using a standardized Excel-based extraction form. Extracted information included author, publication year, country, study design, school setting, number of schools and participants, intervention components, comparison group where applicable, implementation actors, programme duration or scale, implementation strategies, and reported adaptations. Information on theories, models, or frameworks used to guide implementation or evaluation was also extracted. Following Nilsen, theories were understood as explanations of how or why implementation occurs, models as descriptions of implementation processes, and frameworks as structures that organize implementation concepts or determinants [16]. Information on implementation outcomes, implementation determinants, and challenges related to implementation were also extracted. Disagreements were resolved through discussion between the reviewers.

### Data synthesis

Extracted data were summarized descriptively and thematically. Descriptive synthesis was used to report the number of included studies by country, study design, intervention type, and stakeholder group. Implementation outcomes were grouped as acceptability, adoption, feasibility, fidelity, reach, and sustainability. Qualitative, quantitative, and descriptive implementation findings were synthesized separately where direct comparison was not appropriate. Implementation strategies were first extracted from the included studies as reported by the study authors. During synthesis, these strategies were grouped according to their main function, using the ERIC taxonomy as a guiding framework. The categories reflected the strategies identified in the included studies and included training, stakeholder engagement, school-based delivery, community involvement, policy or system integration, and external partnership support. Other ERIC strategies were not reported or could not be clearly identified from the included studies. Barriers and facilitators were synthesized thematically and grouped by resources, infrastructure, governance and coordination, human capacity, acceptability, sustainability, and stakeholder engagement. Findings were presented in narrative form, tables, and figures. Frequencies describe how many included reports mentioned a construct; they were not interpreted as prevalence, importance, or effectiveness across countries or programmes.

## Results

The database search identified 1,985 records. After removal of 1,035 duplicates, 950 records were screened by title and abstract. Screening was conducted using EndNote for record management and an Excel-based screening sheet to record screening decisions. Of the screened records, 889 were excluded because they did not meet the eligibility criteria. Sixty-one articles were sought for full-text assessment, and five full texts could not be retrieved. Fifty-six full-text articles were assessed for eligibility. Of these, 48 were excluded for the following reasons: no implementation-related evidence relevant to the review questions (*n* = 21); not school-based interventions (*n* = 9); not conducted in LMIC settings (*n* = 5); ineligible study design (*n* = 8); and insufficient reporting of the intervention or implementation process (*n* = 5). Eight studies were included in the final review. The selection process is shown in the PRISMA flow diagram (Figure 1).

### Characteristics of included studies

Two studies were qualitative [17,18], two used mixed methods [19,20], two were cross-sectional school assessments [21,22], one was a quantitative implementation analysis [23], and one was a school-based implementation study [24]. Their settings ranged from a single urban school to national or large-scale programmes. Sample size and programme scale also varied substantially, from 9 to 120 schools in the Indian assessments to 4,035 schools and more than 400,000 girls in the Pakistan school lunch programme (Table 1). The included interventions comprised school health and nutrition programmes, weekly iron-folic acid supplementation, school lunch delivery, school meals and gardens, nutrition education, and health-promoting school components. The studies focused on different implementation questions, including stakeholder experiences, delivery processes, adherence, school compliance, feasibility, resource constraints, and continuation (Table 1).

**Table 1.** Characteristics of the included studies (n = 8).

Author, Year	Country and study design	Setting and sample	Target population	Intervention content	Implementation focus	Implementation actors	Framework	Duration/scale	Funding/support source
Shrestha et al. [17]	Nepal; qualitative study	National, district, and school levels; 32 key-informant interviews; number of schools not reported	School children, teachers, parents, school staff, and programme stakeholders	School health and nutrition programme	Implementation processes, perceived effects, coordination, and challenges	Government, education and health authorities, aid agencies, school staff, local organizations, and parents	No formal implementation-science framework explicitly reported	National or programme-level implementation; duration not clearly reported	Government and programme support; details not fully reported
Gosdin et al. [23]	Ghana; quantitative implementation analysis	60 secondary schools; 1,387 adolescent girls	Adolescent girls	School-based iron–folic acid supplementation	Adherence, delivery barriers, facilitators, and coping strategies	Teachers, school staff, and programme implementers	No formal implementation-science framework explicitly reported	Large school-based supplementation programme; student-level tablet consumption reported	Government/programme support; details not fully reported
Pappas et al. [19]	Pakistan; mixed-methods programme evaluation	4,035 government primary girls’ schools; 417,665 girls received feeding; analytical sample 203,116 girls	Schoolgirls, parents, local women, teachers, and community members	Large-scale school lunch programme	Programme delivery, community participation, coverage, funding, coordination, and continuation	Local women, school committees, teachers, government, and NGO/public-private partners	No formal implementation-science framework explicitly reported	Large-scale programme; exact duration should be reported if available from the study	NGO and community support; programme funding reported
Rector et al. [18]	Tanzania; qualitative situation analysis	10 public non-boarding secondary schools; total participant number not reported (school heads, 1–2 teachers and 5–6 students per school, approximately 10 parents at one school, and government stakeholders)	School children, parents, teachers, school administrators, local committees	School meals, school gardens, and nutrition education	Feasibility, stakeholder views, resources, and sustainability constraints	Students, parents, teachers, school administrators, and regional/district stakeholders	No formal implementation-science framework explicitly reported	School-level implementation; duration not clearly reported	Parent/community contributions and local school support
Yuvaraj et al. [20]	India; sequential mixed-methods study	9 schools; 1,281 students and 42 teachers; 2 key-informant and 4 in-depth interviews	School children, teachers, parents, and school-level stakeholders	Health-promoting school activities and school nutrition-related policy implementation	School compliance, barriers, facilitators, and suggested improvements	Teachers, principals, school authorities, and research team	WHO Health Promoting Schools framework	School-level intervention; duration should be checked from the study	Research/programme support; details not fully reported
Joseph et al. [21]	India; cross-sectional study	120 schools; 120 school-level respondents (69 principals/headmistresses and 51 teachers/administrators)	Teachers / school representatives	Health-promoting school and school health/nutrition policy components	School-level compliance across health-promoting domains	Principals, headmistresses, teachers, and school administrators	WHO Health Promoting Schools framework	120 schools assessed	Not reported
Jain et al. [24]	India; implementation study in one school	1 urban school; school staff, teachers, parents, management, and three student groups; total participant number not reported	School children, teachers, parents	Health-promoting school programme	Barrier assessment, adaptation, implementation, monitoring, and behavioural uptake	School management, teachers, staff, parents, students, and research team	Knowledge to Action framework and WHO Health Promoting Schools components	School-based intervention; duration should be checked from the study	Research/intervention support; details not fully reported
Periyasamy et al. [22]	India; cross-sectional survey	61 schools (17 government and 44 private); school heads or representatives interviewed	School children / school representatives	School nutrition services and Health Promoting Schools components	Adherence to school health, nutrition-service, environment, and policy components	School heads, administrators, teachers, and school staff	WHO Health Promoting Schools framework	School-level assessment; number of schools should be checked from the study	Government provision in some schools; details not fully reported

### Implementation strategies

Implementation strategies were taken from the included studies as described by the study authors. We then grouped them by their main purpose, using the ERIC taxonomy as a guide. These groups were based only on the strategies described in the included studies, not on the full ERIC list. The strategies found in this review included training and capacity building, stakeholder and community involvement, school-based delivery, support from external partners, and policy or school system integration.

Training and capacity building were reported in 3/8 studies. In Ghana, teachers were trained to support the delivery of iron–folic acid supplementation to adolescent girls, including distribution procedures and management of implementation challenges [23]. In Pakistan, training was provided to support community-based delivery of the school lunch programme, including the involvement of local women and programme staff [19]. In India, health-promoting school activities were supported through repeated awareness sessions, classroom-based education, and structured behaviour change activities for students [24].

School-based delivery was used across all included studies. Teachers and school staff played an important role in delivering or supporting intervention activities. In Ghana, teachers supported the distribution of iron–folic acid supplements and helped maintain adherence to the supplementation programme [23]. In India, teachers supported health-promoting school activities, awareness sessions, and behaviour change activities [20,24]. In Pakistan and Tanzania, school-level delivery was additionally linked to meal preparation, school committees, parents, or community members [18,19].

Stakeholder and community engagement was reported in 4/8 studies. In Nepal, stakeholder engagement and intersectoral coordination were central to implementation [17]. In Pakistan, community engagement, involvement of local women, and collaboration with non-governmental organisations supported meal planning, purchasing, preparation, and delivery [19]. In Tanzania, parents, teachers, school leaders, and local stakeholders supported meals, gardens, and nutrition education, although dependence on parent contributions also threatened continuity [18]. In India, parent involvement and awareness activities were reported as part of health-promoting school implementation [20,24].

External partnership support was most visible in the Pakistan study, where collaboration with non-governmental organizations supported community mobilization and programme delivery [19]. This support enabled large-scale implementation, but delayed government funding and dependence on external arrangements created implementation and sustainability challenges.

Policy or school-system integration was most evident in the four Indian studies. Yuvaraj et al. [20], Joseph et al. [21], and Periyasamy et al. [22] assessed adherence to WHO Health Promoting Schools components, including nutrition services, school policies, health education, and supportive environments. Jain et al. used the Knowledge to Action framework to tailor and implement health-promoting activities within one school [24].

### Methods and frameworks used to guide implementation

Among the eight included studies, explicit use of theories, models, or implementation frameworks was limited. The WHO Health Promoting Schools framework was used or assessed in the Indian studies by Yuvaraj et al. [20], Joseph et al. [21], Jain et al. [24], and Periyasamy et al. [22]. Jain et al. additionally used the Knowledge to Action framework to develop a health-promoting school intervention [24]. Other studies reported implementation processes, barriers, facilitators, or delivery strategies but did not clearly apply formal implementation science frameworks, such as CFIR, RE-AIM, or Proctor’s implementation outcome taxonomy [17–19,23].

### Implementation outcomes

**Figure 2.**
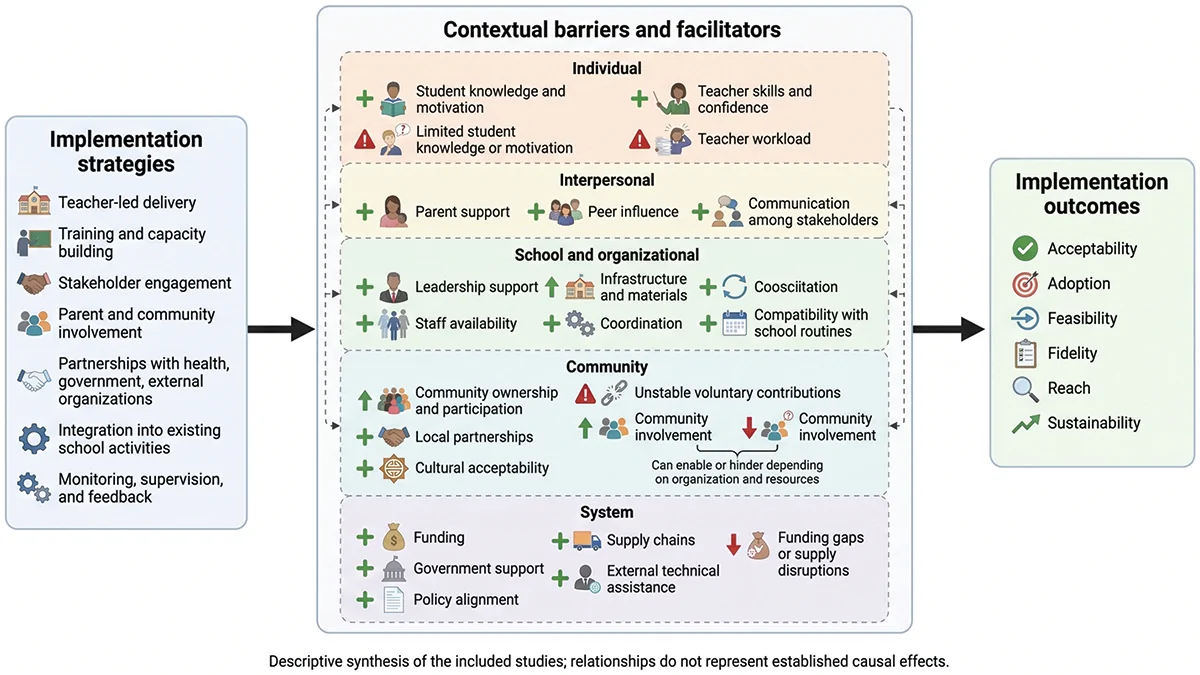
Conceptual summary of the implementation of school-based nutrition interventions in low- and middle-income countries.

Implementation outcomes were labelled and measured (Table 2, Figure 2). Feasibility was reported in 3/8 studies [17,18,24], acceptability in 3/8 [17–19], adoption or closely related constructs in 3/8 [21,23,24], fidelity in 1/8 [23], reach in 1/8 [19], and sustainability-related findings in 3/8 [17–19]. Feasibility and acceptability were usually assessed qualitatively or descriptively through stakeholder views and practical delivery conditions. Feasibility depended on staff time, infrastructure, water, materials, financing, and school-level support [17,18,24]. Acceptability was reflected in stakeholder support, perceived usefulness, community participation, and fit with school or family practices [17,19,20]. The Ghana study additionally described concerns about perceived effects of supplementation, but these data were not directly comparable with the qualitative acceptability findings from other studies [23].

**Table 2.** Implementation outcomes reported in the included studies.

Implementation outcome	Operational definition used in this review	Studies reporting the outcome, n/N	Included studies	Reported indicators
Feasibility	Extent to which the intervention or programme could be delivered within available school resources, time, infrastructure, and personnel	3/8	Jain et al. [24]; Shrestha et al. [17]; Rector et al. [18]	Qualitative/descriptive: practical delivery conditions, staffing, facilities, materials, water, time, and school-level support
Acceptability	Extent to which the intervention was viewed as suitable, satisfactory, or useful by students, parents, school staff, or implementers	3/8	Pappas et al. [19]; Shrestha et al. [17]; Yuvaraj et al. [20]	Qualitative/descriptive: stakeholder support, perceived usefulness, community participation, and fit with school or family practices; supplementation concerns were additionally described by Gosdin et al. (2)
Adoption	Decision or action by schools, teachers, or other implementers to use or deliver intervention components	3/8	Gosdin et al. [23]; Joseph et al. [21]; Jain et al. [24]	Quantitative and descriptive: school/teacher participation, school compliance with programme components, and delivery of behaviour-change activities
Fidelity	Extent to which intervention components were delivered as planned	1/8	Gosdin et al. [23]	Quantitative/descriptive: regularity of teacher-led tablet distribution and adherence to planned supplementation procedures
Reach	Extent to which the intervention reached intended schools, students, or stakeholder groups	1/8	Pappas et al. [19]	Quantitative programme-scale data: 4,035 schools and 417,665 girls received feeding; most other studies did not provide a formal reach denominator
Sustainability	Extent to which programme activities could be continued or institutionalized over time	3/8	Pappas et al. [19]; Shrestha et al. [17]; Rector et al. [18]	Qualitative/descriptive: funding continuity, coordination, external support, and reliance on parent/community contributions; no prospective maintenance measure was used

Adoption, or related concepts such as compliance, implementation level, and behaviour adoption, was reported in three studies. These outcomes were assessed mainly at the school or implementer level rather than only at the student level. In Ghana, school and teacher participation supported delivery of iron–folic acid supplementation. Quantitative data showed that 90% of girls had ever consumed the tablet, while 56% had consumed at least 15 weekly tablets during the school year. Mean intake was 16.4 tablets out of a possible 36 tablets [23]. In India, adoption was described through school-level compliance with health-promoting school components, implementation of school nutrition policies, and student behaviour change activities [21,24].

Fidelity was reported in one study, the Ghana school-based iron–folic acid supplementation programme. In this study, fidelity was assessed through adherence to planned supplementation procedures, including whether teachers delivered the intervention as intended and whether implementation challenges affected regular distribution [23].

Reach was reported most clearly in the Pakistan school lunch programme, which delivered feeding in 4,035 schools to 417,665 girls [19]. Other studies reported sample size, school compliance, service availability, tablet consumption, or stakeholder participation but generally did not provide a formal numerator and denominator for the proportion of eligible schools or students reached. Sustainability-related findings were reported in Nepal, Pakistan, and Tanzania [17–19]. They concerned unstable financing, coordination, external support, and reliance on parent or community contributions. None of the included studies used a formal sustainability measurement tool or followed programmes long enough to assess long-term maintenance.

### Barriers to implementation

Barriers and facilitators were organized at individual, interpersonal, school/organizational, community, and system/policy levels (Table 3). At the individual level, barriers included concerns about side effects, limited knowledge, and difficulty changing nutrition-related behaviours. In the Ghana supplementation programme, concerns about side effects affected the delivery and acceptability of iron–folic acid supplementation [23]. In the Indian behaviour change intervention, students’ ability to adopt healthier behaviours was influenced by cultural food practices and family factors, such as what foods were usually prepared at home, parental support, and access to healthy foods [24].

**Table 3.** Barriers and facilitators to implementation organized by socio-ecological level.

Socio-ecological level	Reported barriers	Reported facilitators	Studies
Individual	Concerns about supplementation effects; limited knowledge; difficulty translating nutrition messages into everyday behaviour; restricted access to healthy foods	Perceived benefits; improved awareness; repeated sensitization; practical and age-appropriate health messages	Gosdin et al.; Jain et al.; Yuvaraj et al.
Interpersonal	Limited parent engagement; family food practices; weak peer support; competing responsibilities and time burden among teachers and staff	Teacher involvement; parent reinforcement; peer and family support; shared responsibility for delivery	Gosdin et al.; Jain et al.; Yuvaraj et al.
School/organizational	Insufficient staff, materials, storage, kitchens, safe water, garden resources, monitoring, or practical curriculum time; uneven delivery across programme components	Training; structured procedures; committed school leadership; existing school space and routines; integration with curricula and school policies	Gosdin et al.; Joseph et al; Periyasamy et al.; Jain et al.; Yuvaraj et al.; Rector et al.
Community	Dependence on voluntary parent/community contributions; difficulty sustaining local resources; inconsistent participation	Community ownership; involvement of local women and school committees; local acceptance and mobilization	Pappas et al.; Shrestha et al.; Rector et al.
System/policy	Unstable or delayed funding; dependence on external partners; unclear responsibilities; weak intersectoral coordination; limited policy support and monitoring	Government or NGO support; public-private partnership; intersectoral engagement; alignment with health-promoting school frameworks	Pappas et al.; Joseph et al.; Periyasamy et al.; Shrestha et al.; Yuvaraj et al.; Rector et al.

At the interpersonal level, barriers included limited parent engagement, weak peer support, and competing responsibilities among teachers and school staff. In Indian health-promoting school studies, lack of manpower, limited time, and teacher workload constrained implementation [24]. In Ghana, teacher time burden and implementation difficulty were important barriers to regular supplementation delivery [23].

At the school or organizational level, barriers included inadequate infrastructure, lack of materials, limited food or supplement supplies, and insufficient facilities. These barriers were reported in studies from Tanzania and India and included inadequate kitchen facilities, limited access to safe water, limited storage space, absence of school gardens, and insufficient physical resources to support school-based nutrition services [17,22]. These limitations affected the feasibility of school meals, school gardens, nutrition education, and school nutrition service delivery.

At the community level, barriers included reliance on parent contributions and difficulty maintaining local support over time, especially in publicly funded or resource-limited school settings. In Tanzania, school meals and related nutrition activities often depended on parent contributions, which limited feasibility and sustainability [17]. In Pakistan, delays in funding and dependence on community-based delivery created challenges for maintaining the school lunch programme [20].

At the system level, barriers included insufficient funding, challenges in programme coordination, unclear roles, and limited monitoring. Resource constraints were reported in studies from Nepal, Pakistan, Tanzania, and India [17–20,22]. Coordination problems were also reported across several studies, including limited intersectoral coordination, unclear responsibilities, and limited monitoring [17,18,20,21]. These problems affected implementation because some school health or nutrition components were not delivered regularly or were implemented differently across schools. These system-level barriers were especially important for sustainability, as programmes were more difficult to maintain when they depended on unstable funding, external partners, or voluntary parent and community contributions.

### Facilitators of implementation

Stakeholder engagement was the most frequently reported facilitator, appearing in 4/8 studies [17–20]. Teachers, parents, school leaders, community members, and local programme actors contributed to intervention delivery, local acceptance, and programme support. In Pakistan, community ownership was particularly important for the school lunch programme [19]. In Nepal and Tanzania, stakeholder support and perceived programme benefits helped strengthen implementation [17,18].

Training and capacity building were reported as facilitators in three studies. Teacher training, structured delivery procedures, awareness programmes, and repeated sensitisation helped improve implementation capacity and stakeholder understanding [19,23,24]. In the Ghana supplementation programme, training and positive perceptions supported delivery [23]. In Pakistan, training supported community-based implementation [19]. In India, structured sessions and awareness activities facilitated behaviour change and health-promoting school implementation [20,24].

The school platform was frequently described as facilitating intervention delivery across the included studies, as it provided regular access to children and adolescents and allowed intervention activities to be incorporated into existing education systems. This was relevant for supplementation [23], school feeding [18,19], nutrition education and school gardens [18], and health-promoting school programmes [20–22,24]. Integration strategies, such as combining school meals with education, linking nutrition activities to school policies, or aligning programmes with health-promoting school frameworks, were reported in studies describing feasibility- and sustainability-related considerations [18,22,24].

## Discussion

This scoping review included eight studies examining the implementation of school-based nutrition interventions in LMICs. The studies covered a diverse set of interventions, including school health and nutrition programmes in Nepal [17], school-based iron–folic acid supplementation in Ghana [23], a large-scale school lunch programme in Pakistan [19], school meals, gardens, and nutrition education in Tanzania [18], and health-promoting school activities, school nutrition policies, and nutrition-related service delivery in India [20–22,24]. The level of implementation evidence varied among these studies. Reach and fidelity were less frequently mentioned than feasibility, acceptability, adoption, and sustainability, indicating that many studies found implementation challenges but failed to measure them using accepted standards of implementation outcomes.

One important finding was that implementation strategies differed by intervention type. Teacher-led delivery and training were most clearly reported in the Ghana iron–folic acid supplementation programme [23] and in Indian health-promoting school interventions [20,24]. By contrast, community engagement and local ownership were more central in school feeding and broader school health programmes. In Pakistan, Pappas et al. described a large school lunch programme that relied heavily on community participation, local women, and non-governmental organization support [19]. In Tanzania, Rector et al. showed that school meals, gardens, and nutrition education depended on parent contributions, school-level support, and local resources [18]. Across the included studies, no single implementation model was consistently reported. Instead, delivery approaches varied by intervention type, responsible actors, and available school-level resources.

The findings also demonstrate that the same contextual factor may function as either a facilitator or a barrier depending on how it is organized and resourced. Community ownership may strengthen local participation, acceptability, trust, and programme delivery, whereas dependence on voluntary parent or community contributions may threaten continuity when these contributions are unstable or substitute for formal programme financing. Programmes should therefore distinguish between community participation and community dependency. The included evidence suggests that community participation may be more sustainable when roles are clearly defined, participation is adequately supported, and core programme costs are covered by stable institutional or governmental resources. However, these conditions should be regarded as implementation considerations rather than established determinants of successful implementation [14].

The limited reporting of reach and fidelity is a notable gap. Reach was most visible in the large-scale Pakistan school lunch programme [19], but most studies did not clearly report how many eligible students were reached or whether participation was sustained over time. Fidelity was most clearly addressed in the Ghana supplementation study [23], where adherence to iron–folic acid supplementation procedures was a central issue. This finding is important because implementation frameworks such as RE-AIM emphasize reach as a key determinant of population-level impact, while fidelity frameworks highlight the need to understand whether an intervention was delivered as intended [25,26]. Without clear reporting of reach and fidelity, it becomes difficult to distinguish between an intervention that is ineffective and an intervention that was not implemented adequately.

Acceptability and feasibility were reported more often, but usually in descriptive ways. Shrestha et al. [17] and Rector et al. [18] reported stakeholder support for school health and nutrition programmes, while Yuvaraj et al. [20] described acceptability and implementation challenges in health-promoting school settings. However, these studies generally did not use standardized acceptability tools. This matters because acceptability includes more than positive perceptions; it may also involve burden, opportunity costs, ethical concerns, and perceived fit with local priorities, as described by Sekhon et al. [27]. Similarly, feasibility was often linked to practical school conditions, such as staffing, time, water access, kitchen facilities, and availability of materials.

Sustainability was another recurring concern. Shrestha et al. [17], Pappas et al. [19], and Rector et al. [18] all reported issues related to long-term continuation, including limited funding, weak coordination, reliance on community contributions, and dependence on external support. Sustainability literature distinguishes between short-term continuation, institutionalization of programme activities, and maintenance of outcomes over time [28–30]. In the included studies, sustainability was usually discussed as a concern rather than measured prospectively. This limits the ability to judge whether school-based nutrition interventions can be maintained after external funding, research support, or initial implementation enthusiasm declines.

The barriers identified in this review were reported across several countries and intervention types. Limited funding, weak infrastructure, inadequate materials, teacher workload, staffing constraints, and weak governance appeared repeatedly. These barriers were reported in different forms across Nepal [17], Ghana [23], Pakistan [19], Tanzania [18], and India [20–22,24]. This indicates that implementation challenges are not isolated problems of individual programmes. Rather, they reflect broader system constraints in LMIC school settings. The CFIR framework helps interpret these barriers because many relate to available resources, organizational readiness, implementation climate, leadership, and external policy or funding environments [31]. However, CFIR was not applied prospectively in most of the included studies and was used in the present review only as an author-driven, post hoc interpretive framework. Accordingly, the identified barriers should not be interpreted as validated determinants of implementation across all LMIC school systems.

Several factors were reported as facilitators across the included studies, including stakeholder engagement, teacher involvement, community ownership, training, awareness activities, and integration into existing school systems. In Pakistan, community ownership and training were central to the school lunch programme [19]. In Ghana, teacher training and positive perceptions were reported as facilitators of supplementation delivery [23]. In Tanzania and Nepal, stakeholder support and perceived programme benefits were reported as facilitators of implementation [17,18]. These findings align with implementation science literature showing that successful implementation requires more than technical intervention design; it also requires planning, engagement, capacity building, and ongoing support [31,32].

The findings of this review are consistent with broader literature on school nutrition and adolescent nutrition. Kristjansson et al. showed that school feeding can improve health and psychosocial outcomes among disadvantaged children, but effects vary by context and programme delivery [33]. Langford et al. reported that health-promoting school approaches can improve health-related outcomes, but whole-school models are complex and require implementation across multiple domains [34]. Salam et al. found that adolescent nutrition interventions can improve nutrition-related outcomes, but delivery platforms, adherence, and implementation quality remain important for success [35]. The present review adds to this literature by showing that, in LMIC school settings, implementation processes are still underreported compared with effectiveness outcomes.

Recent literature provides additional context for these findings. In Malaysia, Majid et al. evaluated the feasibility and outcomes of the MyHEART BEaT school-based nutrition intervention and highlighted the importance of involving school staff, food vendors, parents, and relevant government and community stakeholders [36]. Meshkovska et al. synthesized qualitative evidence on barriers and facilitators to nutrition-related actions in LMIC school settings and identified the importance of resources, organizational readiness, stakeholder needs, external support, and engagement [14]. More broadly, Moore et al. emphasized the value of process evaluations for understanding how complex interventions are delivered, how they produce change, and how context influences implementation [37]. Proctor et al. further showed that implementation outcomes remain unevenly assessed and that relationships between implementation strategies and outcomes are rarely examined [38].

This review has several limitations. First, only eight studies met the inclusion criteria, which limits the strength of the conclusions. Second, the findings were geographically concentrated, with four studies from India and one study each from Nepal, Ghana, Pakistan, and Tanzania. Third, the included studies varied in design, scale, intervention type, and reporting detail. Fourth, many study authors did not clearly report the implementation outcomes, so these outcomes had to be interpreted from the reported findings. For example, some studies described school compliance, service coverage, student tablet consumption, stakeholder support, or programme delivery challenges, but did not label these findings as adoption, reach, acceptability, feasibility, or fidelity. Therefore, some outcomes had to be interpreted from the reported findings, which may have introduced some uncertainty in classification. Finally, formal critical appraisal was not conducted because this was a scoping review; therefore, the review maps the evidence base but does not assess the strength or quality of individual study findings.

In addition, the electronic search was limited to PubMed and Scopus, grey literature was excluded, and only English-language publications were considered. Relevant evidence indexed primarily in education, social science, regional, or implementation-focused databases may therefore have been missed.

The search strategy used implementation-related terminology, which was appropriate for the review questions but may have missed studies that reported implementation-relevant indicators without explicitly labelling them as implementation outcomes. For example, school meal trials may report the number of days meals were delivered, intervention exposure, participation, coverage, adherence, service interruptions, or the proportion of schools receiving programme components without describing these indicators as reach, fidelity, adoption, or feasibility. Implementation information reported only in the full text, but not reflected in titles, abstracts, or keywords, may therefore not have been identified by the search.

More broadly, implementation-related information may be difficult to identify because studies often describe delivery, participation, coverage, or adherence without explicitly referring to these as implementation outcomes. This type of reporting may cause relevant studies to be missed and makes comparisons across interventions more difficult. Clearer reporting would improve future evidence synthesis.

The findings nevertheless point to several practical implications. Future research would benefit from clearer reporting of implementation outcomes, especially reach, fidelity, and sustainability. Process evaluations should be included more often in studies of school-based nutrition interventions, as they can show how programmes are actually delivered, what adaptations are made, and which contextual factors affect implementation. For programme planners and implementers, the reviewed evidence identifies several recurring implementation considerations, including stable financing, adequate infrastructure, trained personnel, clear governance arrangements, and sustained engagement with teachers, parents, communities, and local authorities.

These factors should be regarded as barrier-informed priorities rather than as proven requirements, because the included studies did not compare their effectiveness or demonstrate that they caused improvements in implementation outcomes. Future evaluations should examine prospectively how specific implementation strategies affect reach, adoption, fidelity, feasibility, acceptability, sustainability, and cost.

In conclusion, several of the included studies reported school-based nutrition interventions as feasible or acceptable within their specific settings while also identifying implementation barriers such as unstable funding, limited infrastructure, staff workload, poor coordination, limited monitoring, and dependence on external or parent contributions. It would be easier to determine which interventions can be implemented at scale, modified for local contexts, and maintained over time if implementation processes were better documented. These findings should not be generalized to all LMIC school systems. The available evidence does not establish which interventions are scalable, transferable across contexts, or sustainable over time; instead, it highlights the need for prospective evaluation of adaptation, scale-up, institutionalization, and long-term maintenance.

## Supplementary Material

Supplementary File S1.docx

PRISMAScRFillableChecklist.pdf
