# Supplementary material for: Implementing school-based nutrition interventions in low- and middle-income countries: a scoping review of implementation strategies, frameworks, and outcomes
Source: Glob Health Action. 2026 Sep 8;19(1):2727312. doi: 10.1080/16549716.2026.2727312 (PMC13556112; doi:10.1080/16549716.2026.2727312)
Supplement: Supplementary file 2 [file ZGHA_A_2727312_SM4119.docx]

**PubMed**

(

(school*[tiab] OR "school-based"[tiab] OR "primary school"[tiab] OR

"secondary school"[tiab] OR "educational institution"[tiab])

AND

(child*[tiab] OR adolescen*[tiab] OR student*[tiab] OR pupil*[tiab] OR youth[tiab])

)

AND

(

(nutrition*[tiab] OR "school meal*"[tiab] OR "school feeding"[tiab] OR

"school lunch"[tiab] OR "school snack*"[tiab] OR "food supplementation"[tiab] OR

"food fortification"[tiab] OR micronutrient*[tiab] OR "nutrition education"[tiab] OR

"healthy eating"[tiab] OR "dietary intake"[tiab] OR "food habit*"[tiab] OR

"food environment"[tiab] OR "canteen polic*"[tiab] OR "food polic*"[tiab])

)

AND

(

(implement*[tiab] OR "implementation science"[tiab] OR "program* delivery"[tiab] OR

"programme delivery"[tiab] OR "scale-up"[tiab] OR "scaling up"[tiab] OR

sustainab*[tiab] OR adopt*[tiab] OR fidelit*[tiab] OR

"implementation strateg*"[tiab] OR "delivery strateg*"[tiab] OR

"implementation framework*"[tiab] OR "implementation model*"[tiab] OR

"implementation theor*"[tiab] OR CFIR[tiab] OR

"Normalisation Process Theory"[tiab] OR NPT[tiab] OR "RE-AIM"[tiab] OR

"Theoretical Domains Framework"[tiab] OR TDF[tiab] OR

"logic model"[tiab] OR "process evaluation"[tiab] OR

"mechanism* of action"[tiab] OR "implementation outcome*"[tiab] OR

acceptab*[tiab] OR feasibilit*[tiab] OR reach[tiab] OR uptake[tiab] OR

barrier*[tiab] OR facilitator*[tiab] OR "Proctor"[tiab] OR

determinant*[tiab] OR "contextual factor*"[tiab] OR

"knowledge translation"[tiab] OR "evidence-based practice"[tiab])

)

AND

(

("low-income countr*"[tiab] OR "middle-income countr*"[tiab] OR

"low- and middle-income"[tiab] OR LMIC[tiab] OR LMICs[tiab] OR

"developing countr*"[tiab] OR "resource-limited"[tiab] OR

"resource-constrained"[tiab] OR Africa[tiab] OR "sub-Saharan Africa"[tiab] OR

Asia[tiab] OR "South Asia"[tiab] OR "Southeast Asia"[tiab] OR

"Latin America"[tiab] OR Nepal[tiab] OR Bangladesh[tiab] OR Ethiopia[tiab] OR

Uganda[tiab] OR Kenya[tiab] OR Ghana[tiab] OR Tanzania[tiab] OR Nigeria[tiab] OR

India[tiab] OR Pakistan[tiab] OR Indonesia[tiab] OR Cambodia[tiab] OR

Vietnam[tiab] OR "Developing Countries"[MeSH])

)

# Scopus

(

TITLE-ABS-KEY(school* OR "school-based" OR "primary school" OR

"secondary school" OR "educational institution")

AND

TITLE-ABS-KEY(child* OR adolescen* OR student* OR pupil* OR youth)

)

AND

(

TITLE-ABS-KEY(nutrition* OR "school meal*" OR "school feeding" OR

"school lunch" OR "school snack*" OR "food supplementation" OR

"food fortification" OR micronutrient* OR "nutrition education" OR

"healthy eating" OR "dietary intake" OR "food habit*" OR

"food environment" OR "canteen polic*" OR "food polic*")

)

AND

(

TITLE-ABS-KEY(implement* OR "implementation science" OR

"program* delivery" OR "programme delivery" OR "scale-up" OR

"scaling up" OR sustainab* OR adopt* OR fidelit* OR

"implementation strateg*" OR "delivery strateg*" OR

"implementation framework*" OR "implementation model*" OR

"implementation theor*" OR CFIR OR

"Normalisation Process Theory" OR NPT OR "RE-AIM" OR

"Theoretical Domains Framework" OR TDF OR "logic model" OR

"process evaluation" OR "mechanism* of action" OR

"implementation outcome*" OR acceptab* OR feasibilit* OR

reach OR uptake OR barrier* OR facilitator* OR Proctor OR

determinant* OR "contextual factor*" OR

"knowledge translation" OR "evidence-based practice")

)

AND

(

TITLE-ABS-KEY("low-income countr*" OR "middle-income countr*" OR

"low- and middle-income" OR LMIC OR LMICs OR "developing countr*" OR

"resource-limited" OR "resource-constrained" OR Africa OR

"sub-Saharan Africa" OR Asia OR "South Asia" OR "Southeast Asia" OR

"Latin America" OR Nepal OR Bangladesh OR Ethiopia OR Uganda OR

Kenya OR Ghana OR Tanzania OR Nigeria OR India OR Pakistan OR

Indonesia OR Cambodia OR Vietnam)

)
